# An EAACI task force report: recognising the potential of the primary care physician in the diagnosis and management of drug hypersensitivity

**DOI:** 10.1186/s13601-018-0202-2

**Published:** 2018-05-10

**Authors:** I. Doña, J. C. Caubet, K. Brockow, M. Doyle, E. Moreno, I. Terreehorst, M. J. Torres

**Affiliations:** 1 Allergy Unit (Pavilion C), Regional University Hospital of Malaga, UMA, IBIMA, National Network ARADyAL, Plaza del Hospital Civil, 29009 Malaga, Spain; 2 Department of Child and Adolescent, Medical School of the University of Geneva, University Hospitals of Geneva, Geneva, Switzerland; 3 Department of Dermatology and Allergy Biederstein, Technische Universität München, Munich, Germany; 4 Indigo Medical, Saint Helier, Jersey; 5 Allergy Service, University Hospital of Salamanca, National Network ARADyAL, Salamanca, Spain; 6 Biosanitary Institute of Salamanca, Salamanca, Spain; 7 Department of Biomedical and Diagnostics Sciences, Salamanca Medical School, Salamanca, Spain; 8 Department of ENT, Academisch Medisch Centrum, Amsterdam, The Netherlands

**Keywords:** Anaphylaxis, Betalactam, Drug allergy, Drug provocation test, Exanthem, Hypersensitivity, Non-steroidal anti-inflammatory drugs, Skin test, Urticaria

## Abstract

Adverse drug reactions include drug hypersensitivity reactions (DHRs), which can be immunologically mediated (allergy) or non-immunologically mediated. The high number of DHRs that are unconfirmed and often self-reported is a frequent problem in daily clinical practice, with considerable impact on future prescription choices and patient health. It is important to distinguish between hypersensitivity and non-hypersensitivity reactions by adopting a structured diagnostic approach to confirm or discard the suspected drug, not only to avoid life-threatening reactions, but also to reduce the frequent over-diagnosis of DHRs. Primary care physicians are often the first point of contact for the sufferer of a reaction, as such they have a key role in deciding whether to discard the diagnosis or send the patient for further investigation. In this review, we highlight the importance of diagnosing DHRs, analysing in detail the role of primary care physicians. We describe the common patterns of DHRs and signs of its progression, as well as the indications and contraindications for referring the patient to an allergist. The diagnostic process is described and the possible tests are discussed, which often depend on the drug involved and the type of DHR suspected. We also describe recommendations regarding the avoidance of medication suspected to have caused the reaction and the use of alternatives.

## Background

**Fig. 1:**
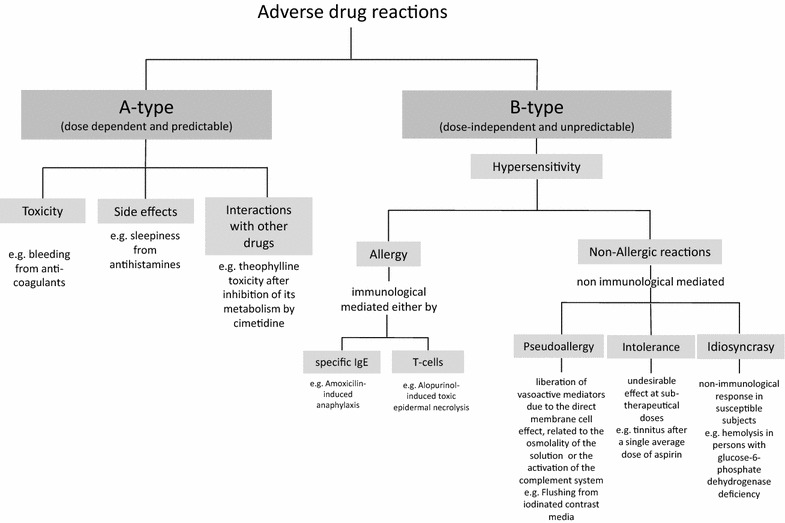
Classification of ADRs

According to the World Health Organization, adverse drug reactions (ADRs) are considered as any noxious and unintended response to a medication that occurs at normal doses used for prophylaxis, diagnosis and/or treatment [1]. ADRs can be classified as A-type and B-type reactions [2] (Fig. 1). *A*-*type reactions* are the most common (70–80%), and are a consequence of the pharmacological action of the drug, occuring in otherwise normal patients. They are dose dependent and predictable [3]. *B*-*type reactions* are less common and are considered dose-independent, unpredictable and unrelated to the pharmacological effects of the drug when taken at normal dosage [3]. They include drug hypersensitivity reactions (DHRs) that usually affect subjects with prior genetic predisposition [4, 5]. DHRs can be immunologically mediated, either by drug-specific antibodies or T-cells, or non-immunologically mediated [6]. The term allergy should only be used to describe reactions for which an immunological mechanism has been demonstrated [6]. The primary care physician plays a key role in determining which patients may have suffered a DHR; in this case, it is important to refer them for specialist evaluation, because there is mounting evidence indicating that inaccurate DHRs diagnosis brings negative repercussions for the patient [7]. However, in many cases the primary care physician will label the majority of patients as allergic without further enquiry, leading to problems related to overdiagnosis. The aim of this review is to highlight the importance of correctly diagnosing DHRs, suggesting a structured diagnostic approach for primary care physicians to follow when a DHR is suspected, recognition of the signs that indicate a reaction requires urgent management, and emphasizing the criteria for referring patients experiencing DHRs to specialists.

## Methods: search strategy

Electronic literature searches of the MEDLINE and EMBASE databases were performed using the following key words: adverse drug reaction, drug allergy, hypersensitivity, anaphylaxis, urticaria, exanthema, betalactam, non-steroidal anti-inflammatory drugs, drug provocation test, primary care, and general practitioner. Each article was reviewed for suitability and a consensus was reached among the authors regarding the recommendations for when a patient should be referred to an allergist for evaluating a suspected DHR.

### Classifying DHRs

The classification of DHRs is difficult as for many drugs and clinical presentations the underlying mechanisms are poorly understood. They can be classified based on the delay between the last drug administration and the onset of the reaction as either an *immediate reaction*, when occurring up to 1 h after the drug intake, or a *non*-*immediate reaction*, when occurring after more than 1 h [6]; in reality non-immediate reactions can occur several days after treatment. *Immediate reactions* are mainly induced by an IgE-mediated mechanism and *non*-*immediate reactions* are often specific T cell mediated, although other mechanisms can be involved [6]. However, this chronological classification has limitations due to the arbitrary cut-off point of 1 h: firstly, the exact occurrence of initial signs of a drug allergy might be hard to pinpoint in the clinical history. Secondly, the route of administration can influence the time interval in which the reaction starts, e.g. antibiotics can elicit severe anaphylaxis within a few minutes after parenteral administration, but can take up to 1–2 h to do so after oral intake. Thirdly, drug metabolites may take some hours to be formed and therefore an IgE-mediated immediate reaction can start much later than 1 h after drug intake. Finally, cofactors such as exercise, food intake, alcohol and co-medications can speed up or slow down the onset or progression of a reaction [8]. Moreover, there are DHRs induced by non-immunological mechanisms such as cross-reactivity induced by non-steroidal anti-inflammatory drugs (NSAIDs) that are caused by changes to pharmacological pathways e.g. inhibition of cyclooxygenase [9]. Cross hypersensitivity represents the majority of DHRs induced by NSAIDs compared to specific immunological mediated-reactions (76 vs. 24%) [9].

### The importance of diagnosing DHRs

DHRs are often self-reported and unconfirmed. This is a frequent problem in daily clinical practice and has a considerable impact on prescription choices and patient health. In fact, many more patients suspect they have a DHR than can be confirmed, indicating the importance of an accurate diagnosis of DHRs, which will facilitate appropriate treatment options and preventive measures.

In a tertiary care hospital, 35.5% of admitted patients reported at least one DHR. The main categories were penicillin (12.8%), sulphonamides (7.4%), opiates (6.8%) and NSAIDs (3.5%) [10]. This contrasts with the results of a retrospective study by Blumenthal et al., who used data from 62,719 patients aged 18 years and older taken from a longitudinal database in a large health care system from the Boston area of the United States, who were evaluated for ADRs [11]. Of these, 1035 patients (1.7%) had an ADR, of which only 189 presented symptoms suggestive of a DHR. In primary care, it was recently reported in a retrospective study by Salden et al. that 2% (n = 163) of patients had a diagnosis of reported betalactam (BL) allergy [12]. When physician notes were explored further, of these 163 patients 25 (15.3%) had insufficient information to evaluate whether they had symptoms suggestive of a DHR, 8 (4.9%) had a probable DHR and, 111 (68.1%) a possible DHR. A limitation of this study was that a follow-up using skin test and/or provocation was not performed. In a recent study by Abrams et al., 306 patients with a suspected allergy to BL antibiotics referred from community primary care providers (family physicians or pediatricians) were evaluated [13]. In total, 296 patients underwent an oral challenge: 2 (0.7%) patients had a type 1 reaction and 4 (1.3%) patients had a delayed reaction. In 6 (1.9%) patients no challenge was performed because history was suggestive of a serious delayed reaction. Therefore, there was a low rate of ‘true’ BL hypersensitivity and 96% of patients initially suspected to be allergic were advised that they could safely use BL antibiotics in the future.

These unconfirmed diagnoses can have major consequences for further treatment. As the study of Macy has shown [14], patients with a perceived BL allergy are at higher risk for an infection with methicillin-resistant *Staphylococcus aureus* or vancomycin-resistant Enterococcus; furthermore, they are readmitted more often. They also experience longer hospital stays as well as higher morbidity and even mortality. In perceived NSAID allergy, patients either have to endure pain and inflammation, or move to alternatives such as opioids or paracetamol or, if salicylates are prescribed as an antithrombotic, other agents such as clopidogrel may need to be used; these alternatives all potentially present more serious side effects. It is also the case that patients with asthma are frequently denied NSAIDs unnecessarily, due to perceived risk. This problem is further compounded by the fact that it can be difficult to diagnose a drug allergy based solely on clinical data. As Caubet et al. [15] have shown in a pediatric population, only a minority of the children with skin symptoms could be proven to be truly BL allergic.

### Knowledge gaps and the importance of primary care physicians

The increasing incidence of ADRs has led to a marked rise in consultations with primary care physicians. If the reaction is A-type, it is likely that the primary care physician will be able to manage it within the practice, however if it is a B-type reaction, it will require a structured diagnostic process and potentially further referral to allergy specialists [16]. It has been reported that only 38.5% of primary care physicians felt reasonably competent in DHR diagnosis and 63% expressed a high or medium need for further education [17]. However, over 60% of primary care physicians routinely recommend specific diagnostic tests such as blood or skin tests, despite little or no basic knowledge in allergology [18], and without competence in the management of DHR, which is difficult to attain [19]. This clear gap in knowledge has led to recognised deficits in managing DHRs [20] and conversely, many unnecessary referrals [21, 22]. To improve this situation, it is imperative that levels of knowledge and skills in primary care are improved. There is a clear need for sustained and consistent accessible educational programmes [23]. There is a clear demand for such programmes, as surveys have suggested that 29–96% of primary care physicians have expressed interest in future training in this field [18, 24]. Online guidelines, courses and workshops have been specified as the preferred learning modalities [17].

When analysing the role of primary care physicians in the detection and diagnosis of DHRs and the identification of the culprit drug, it is important to answer the question of what primary care physicians should know about DHRs. Primary care physicians needs to be able to: (1) recognize the common patterns of DHRs (Table 1); (2) identify signs indicating a possibly severe DHR (Table 2); (3) distinguish DHRs from A-type adverse effects; (4) know what important information must be retrieved from the clinical history (Table 3), (5) adequately inform the patient about risk and drug avoidance, and (6) perform accurate recording and coding of this reaction in the patient record.

**Table 1:** Symptoms of the acute phase of the reactions induced by drugs Modified from the NICE Clinical Guideline CG183 [59]

Type of reaction	Clinical entity	Symptoms
Immediate: onset usually < 1 h after drug exposure (previous exposure not always confirmed)	Anaphylaxis	Erythema, urticaria or angioedema and Hypotension and/or bronchospasm
	Urticaria or angioedema without systemic features	Wheals Angioedema
	Exacerbation of asthma	Dyspnea Cough Chest tightness Wheezing
Non-immediate without systemic involvement: onset usually 6–10 days after first drug exposure or within 3 days of second exposure	Exanthema-like	Widespread red macules or papules
	Fixed drug eruption	Single or multiple erythematous plaques that arise at the same site after the intake of the same drug and that resolve leaving post-inflammatory hyperpigmentation
Non-immediate reactions with systemic involvement: onset usually 2–6 weeks after first drug exposure or within 3 days of second exposure.	Drug reaction with eosinophilia and systemic symptoms (DRESS) or drug hypersensitivity syndrome (DHS)	Widespread red macules, papules or erythroderma Fever Lymphadenopathy Liver dysfunction Eosinophilia
	Toxic epidermal necrolysis or Stevens–Johnson syndrome	Painful rash and fever Mucosal or cutaneous erosions Vesicles, blistering or epidermal detachment Red purpuric macules or erythema multiforme
	Acute generalized exanthematous pustulosis (AGEP)	Widespread pustules Fever Neutrophilia
Other common disorders rarely caused by drug allergy	Eczema Hepatitis Nephritis Photosensitivity Vasculitis

**Table 2:** Signs indicating the possible severe progression of a DHR

Type of reaction	Signs indicating a severe reaction Referral advised	
Immediate reaction (anaphylaxis)	Sudden onset of extensive pruritus (in particular palmoplantar and scalp) Flush on face and neck with conjunctivitis and rhinitis Angioedema of the oral mucosa (in particular pharynx and larynx) Severe urticaria Dyspnea and bronchospasm (especially in asthmatics) Hypotension	
Delayed reaction	Cutaneous signs	Centrofacial edema (diffuse erythematous swelling) Involvement of large body surfaces or erythroderma Painful skin Atypical target lesions Nikolsky sign positive^a^ Erosive stomatitis Mucositis (especially if affecting more than one mucosal area) Hemorrhagic necrotizing lesions Purpura
	Signs indicating internal organ involvement	Sudden onset of high fever (> 39 °C), otherwise unexplained Disseminated lymphadenopathy Arthralgias and arthritis

^a^It is a clinical dermatological sign characterized by detachment of the epidermis when rubbing the skin with weak or moderate pressure. The sign is positive if when exerting a slight pressure there is detachment of the skin, leaving wet and red areas

**Table 3:** Data that should be recorded by the primary care physicians in the clinical history of patients with suspected drug allergy

Data that should be recorded in the case of a suspected DHR	
Date of the reaction	
The name of the incriminated drug and reason for prescribing	
The number of doses taken before the reaction occurred	
Time interval between the last dose of drug intake and the onset of the reaction	
The nature and detailed description of the symptoms of the reaction	
The treatment needed to resolve the reaction	
The time for recovery	
Other medications taken (both at the time of the reaction and other chemically related drugs after the reaction)	
Underlying conditions (such as chronic urticaria/chronic rhinosinusitis)	

All the important characteristics of the reaction should be recorded in the clinical history and once a predictable A-type adverse reaction is excluded and a B-type DHR is suspected the following must be performed: (1) inform the patients about what drugs to avoid until the diagnosis is finally confirmed, listing safe non-cross-reactive alternatives and explaining future tests to be done if necessary; (2) know when to refer the patient to the specialist for further allergological testing as soon as possible as described below in order to discard or confirm the DHR; (3) retain the diagnosis of DHR and inform patients and healthcare professionals about it until the end of diagnostic testing, in order to reduce the chance of severe reactions due to subsequent exposure. However, it is crucial to ensure that this diagnosis label is updated as soon as the testing has been completed, in order to ameliorate the problem of overdiagnosis. When possible, details of drug reactions should be included in all correspondence regarding the patient and ideally on hand-written or printed prescriptions; likewise, should the situation change and should the patient be later confirmed as non-allergic, this should be reflected appropriately.

### When to refer to the allergist

Several factors must be taken into account when dealing with a suspected DHR. The most important include clinical manifestations (whether compatible with a DHR, see Table 1), chronology of the symptoms (previous exposure, the temporal relationship between the administration of the drug and the onset of symptoms, the effect of stopping the incriminated drug and the time to recovery), other medications taken (both at the time of the reaction and other chemically-related drugs after the reaction), and underlying conditions (such as chronic spontaneous urticaria (CSU) or chronic rhinosinusitis, which can be aggravated by the intake of certain drugs such as aspirin and other NSAIDs).

As stated above, DHRs are often classified according to the time interval between intake and reaction onset as immediate and non-immediate reactions [6]. Regarding symptoms, immediate reactions usually manifest as isolated urticaria, angioedema, rhinitis, conjunctivitis, bronchospasm, gastrointestinal symptoms (nausea, vomiting, diarrhea), or anaphylaxis with or without cardiovascular collapse (anaphylactic shock) [25]. Non-immediate reactions often affect the skin, with variable cutaneous symptoms. They usually appear as delayed urticarial and maculopapular eruptions. The clinical manifestation of urticaria is indistinguishable in both immediate and non-immediate reactions, and the only parameter that can differentiate them is the time interval between drug intake and the onset of the reaction. In these cases it is very difficult to differentiate between immediate and non-immediate reactions, as this chronological classification has limitations as described above. Non-immediate reactions can also appear as more heterogeneous and less frequent clinical entities such as fixed drug eruptions, vasculitis, blistering diseases (such as toxic epidermal necrolysis (TEN), Stevens–Johnson syndrome (SJS) and generalized bullous fixed drug eruptions), drug-induced hypersensitivity syndrome (DHIS)/drug reaction with eosinophilia and systemic symptoms (DRESS), acute generalized exanthematous pustulosis (AGEP) and symmetric drug-related intertriginous and flexural exanthemas (SDRIFE). Internal organs can be affected, either alone or with cutaneous symptoms; these include hepatitis, renal failure, pneumonitis, anemia, neutropenia, and thrombocytopenia [26].

Any drug can cause a DHR. However, some drugs are more likely to be associated with certain types of reactions and clinical presentations. NSAIDs, especially ibuprofen [27] and BL, and amoxicillin [28, 29] are the most common culprit drugs, inducing immediate reactions such as urticaria and anaphylaxis. Furthermore, there are other drugs such as neuromuscular blocking agents (NMBA) that have been classically considered as the group that most frequently causes anaphylaxis [30]. In addition to these drugs, in recent years fluoroquinolones such as moxifloxacin and proton pump inhibitors such as lansoprazol have been increasingly reported as eliciting anaphylaxis [31–33]. Antiepileptic drugs, antibiotics and allopurinol are considered the most frequent triggers of non-immediate cutaneous adverse reactions, particularly DHIS/DRESS, and can cause reactions several weeks after beginning administration [34]. Concerning other drugs such as iodinated contrast media (ICM), reports of immediate reactions have decreased, however reports of non-immediate reactions have increased [33, 35].

It is also important to identify specific medical conditions that may play a role in DHRs, such as CSU/chronic rhinosinusitis, autoimmune or infectious diseases such as human immunodeficiency virus infection [36]. NSAIDs or Aspirin-exacerbated respiratory disease is an entity per se within the large spectrum of NSAID hypersensitivity in patients with asthma/rhinitis [37]. Up to one-third of the patients with CSU experience exacerbation of their skin symptoms upon ingestion of aspirin and other NSAIDs [9]. The degree of sensitivity may show temporary fluctuations related to the activity of their underlying CSU and sensitivity may even disappear in some patients, therefore NSAID tolerance should be reassessed as appropriate [38, 39].

The indications and contraindications for referring a patient to a specialist are shown in Table 4. When these conditions are met, and a DHR is suspected, it is crucial to refer the patient to an allergy unit.

**Table 4:** Indications for referring a patient to an allergist for evaluating a suspicion of DHRs

Referral mandatory	Referral recommended	Referral not indicated
When there is a history of severe DHR for any drugs such as anaphylaxis or severe non-immediate cutaneous reaction to a drug (e.g. drug reaction with eosinophilia and systemic symptoms (DRESS), Stevens-Johnson syndrome, toxic epidermal necrolysis (TEN), in order to confirm the culprit and protect the patient from future reactions When there is a history of DHRs and the drugs incriminated are local or general anesthetics Patients with a suspected DHR to BL antibiotics who are likely to need these antibiotics in the future (e.g. splenectomy recurrent bacterial infections or immune deficiency, etc.) Patients with a confirmed or suspected DHR to non-BL antibiotics (e.g. macrolides, quinolones) Patients with a suspected DHR to NSAIDs and who are likely to require therapy with this group of drugs in the future For others drugs, when they are required depending on an individual medical need	Patients with a suspected non-severe DHR to BL antibiotics. Although at the moment of the reaction the patient may have no condition that requires BL antibiotics, they are among the most commonly prescribed antibiotics and they are likely to be prescribed in future Patients with a suspected non-severe DHR to NSAIDs. Although at the moment of the reaction the patient may have no condition that requires NSAIDs, they are among the most commonly prescribed drugs and they are likely to be prescribed in future.	Non-compatible symptomatology for a DHR, for example side effects such as gastrointestinal symptoms with antibiotics or dyspepsia after ASA intake Non-compatible chronology Reactions without having taken drugs Subjects without a prior history of a DHR, in particular in preoperative settings

It is important to take into account that the time interval between the suspected DHR and the allergological study can affect the outcome of the tests. A loss of sensitivity to drugs over time has been reported for IgE-mediated reactions, e.g. in subjects with immediate allergic reactions to BLs [40], dypirone [41] and NMBA [42]. After a time interval of more than 6–12 months, some drug tests may already give negative results. Therefore, the allergy workup should ideally be carried out 4–6 weeks after the complete resolution of all clinical signs and symptoms. Moreover, when the time interval between the reaction and the allergy assessment is longer, history is often less reliable and there is often a lack of accurate information: the chronology is imprecise, the clinical manifestations are heterogeneous and the exact name of drug or corrective treatment may not be recalled by the patient, making drug causality assessment more difficult to ascertain [43].

### The diagnostic procedure: what happens in specialist centres

**Fig. 2:**
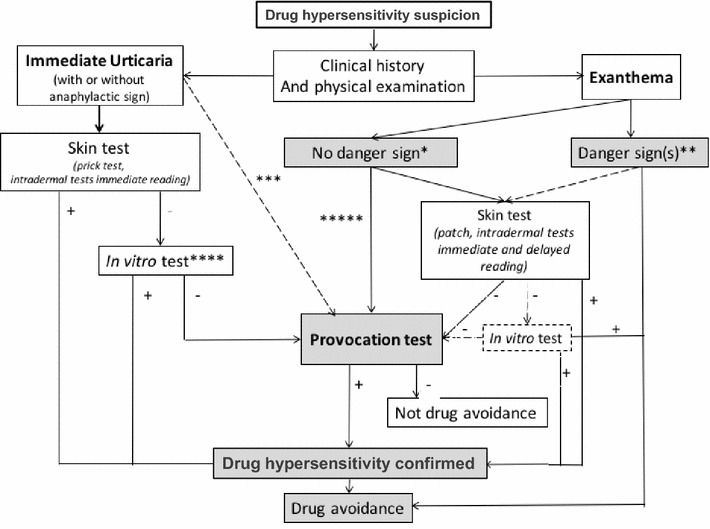
Diagnostic procedures for the diagnosis of DHRs. *Non severe uncomplicated exanthemas. **This category include more severe exanthemas, such as those with high extent and density of skin lesions and long duration, complication or danger signs. It includes also acute generalized exanthematic pustulosis, drug reaction with eosinophilia and systemic symptoms, Stevens Johnson Syndrome or toxic epidermal necrolysis. In specific cases, skin tests may be considered for identification of culprit among several used drugs. ***For NSAID and non-BL antibiotics, the diagnostic value of skin tests is not well defined. In case of isolated urticaria, a DPT can be performed directly. ****Validated *in vitro* tests recommended before skin tests if history of severe reaction or if skin tests are not possible or refused. They may confirm hypersensitivity only together with convincing history and/or other tests. Practically, specific IgE are mainly used for suspicion of hypersensitivity to BL antibiotics. ******In the pediatric population, it has been shown that a drug provocation test can be performed directly, without skin test before, in children with a non severe uncomplication exanthemss. If there is any doubt, skin tests should be performed before drug provocation test

In most European countries the diagnostic assessment takes place in specialized centers and is adapted depending on the drug involved and the type of allergic reaction suspected (e.g. immediate or non-immediate) (Fig. 2). Therefore, the accurate description of the reaction recorded by the primary care physician is of great importance, especially if recorded in a standardized format [44]. In particular, identification of the signs indicating a potentially severe DHR is crucial (Table 2) [6, 45].

The diagnosis approach differs for immediate and non-immediate reactions. For immediate reactions, it normally includes skin prick tests and immediate-reading intradermal tests (IDTs). Non-irritating concentrations for skin tests have been determined for a large range of drugs (e.g. BLs, perioperative drugs, heparins, platinum salts, and ICM) [46]. By using these non-irritating concentrations, a positive skin test suggests that the patient is at significant risk of an IgE-mediated reaction to the incriminated drug [46]. Regarding in vitro tests to diagnose immediate reactions, specific IgE have been used for a long time for a limited number of drugs (e.g. some BL antibiotics, NMBA and chlorhexidine) [6, 47]. Their diagnostic value depends on the drug, but specific IgE is generally less sensitive compared to skin tests. They are the first line in patients with a history of severe immediate anaphylaxis, due to the potential risk of systemic reactions during skin testing [6]. Basophils activation tests (BATs) have received increasing interest recently, and although they are not available in all centers nor standardized for all drugs, BATs have shown to be useful and are recommended for diagnosing IgE-mediated reactions to BLs, NMBA, pyrazolones, fluoroquinolones and ICM [47]. Both immunoassays and BAT are recommended in high-risk patients before drug provocation tests (DPTs) and even skin tests [47].

DPTs involve the controlled administration of a drug under medical surveillance. They are widely considered to be the gold standard to establish or discard the diagnosis of hypersensitivity to a certain substance [48]. As the negative predictive value of other tests (both in vivo and in vitro tests) is not 100%, DPTs should be performed to formally exclude an immediate allergic reaction after negative skin tests and/or specific IgE or BAT [6]. Moreover, within NSAID DHRs, NSAID cross-hypersensitivity comprises reactions not caused by specific immunological mechanisms but through changes to pharmacological pathways e.g. inhibition of cyclooxygenase, therefore the DPT is currently the only diagnostic test available [9]. This is crucial as cross-hypersensitivity represents the majority of DHRs induced by NSAIDs [9]. Note that DPTs should be performed ideally 2 months after the initial reaction, in a secure environment by a trained team.

Regarding diagnosis of non-immediate reactions, delayed-reading IDTs and/or patch tests are generally recommended [6]. These tests have been studied for a limited number of drugs (mainly antibiotics) and data are lacking regarding the standardization of concentrations and vehicles for the majority of other drugs. In children, it has been shown that in patients developing a benign exanthema (without any danger signs), DPTs can be performed without prior skin testing [15, 49]. Although these reactions are not DHRs in nature, they need to be studied before prescription of subsequent antibiotics. It is important to note that the clinician should be certain that the child does not show any signs associated with a potential severe reaction, highlighting the importance of an accurate description of the reaction during the acute phase in medical records. For patients with a history of severe reactions (such as TEN, SJS, DIHS/DRESS and AGEP), the use of a DPT is contraindicated. If the use of the drug is essential, an allergy workup can be discussed. Due to the potential risk of triggering a severe systemic reaction, the diagnostic procedure will start with a patch test and if negative, IDTs can be performed, starting with the highest dilutions [6]. It should be made clear that the true diagnostic value of skin tests for severe non-immediate reactions is not known, as confirmatory DPTs have rarely been performed for ethical reasons.

Regarding in vitro tests to diagnose non-immediate reactions, the lymphocyte transformation test and lymphocyte activation test have been suggested to be useful, however their diagnostic value remain unclear [47]. Further studies are needed before they can be recommended in international guidelines [6].

### Avoidance and alternative drugs

When a patient with a history of DHRs attends a primary care physician office, the standard procedure for the vast majority of patients is to stop the suspected medication reported to have caused the reaction, and, if the patient is still in need of drug therapy, to give a non-cross-reactive alternative drug [50]. Other options, such as treating through or desensitization are only used in certain specific situations such as patients with coronary artery disease and allergy to NSAIDs who need aspirin for platelet antiaggregation treatment. They should normally be supervised by an allergist experienced in DHRs [51].

In patients who report having taken multiple drugs, a very important decision is which drugs to stop and which to continue, as alternative drugs are often not as effective and/or are associated with a higher level of predictable adverse reactions. To answer this question, the exact chronology is crucial. As shown in Table 1, drugs that have been continuously taken for months are not suspected to have caused a new drug allergic reaction. There is a certain time window between beginning drug intake and elicitation of first symptoms depending on the DHR symptoms. This holds true for the manifestations listed in Table 1, with the exception of ACE (angiotensin-converting-enzyme) inhibitor induced DHRs such as angioedema, which do not follow this rule and can develop after months or even years of intake. Another exception is when a drug is discontinued and retaken, which theoretically may cause a new sensitization and reaction upon re-administration. Otherwise, a drug causing anaphylaxis is usually one that has been given within 1 h, sometimes (especially for NSAIDs) within up to 6 h before the reaction [6]. A repeat fixed drug eruption may arise 30 min to 6 h after intake of the offending drug. For non-immediate benign exanthemas, elicitation of a reaction takes more than 6 h, usually around 10 days after initial sensitization or 2–3 days after renewed intake (Table 1). In patients with multiple drug intake, it is important to record the dates and periods of intake for all drugs taken within the last few (for DRESS up to 8) weeks in order to get a better overview of the possible culprits. Normally, only the drugs within the possible time window need to be withdrawn. However, when patients react to several different drugs given in succession it has to be considered that even after the discontinuation of a drug, a deterioration of the exanthema should be expected, For example, in a benign exanthema occurring 10 days after introduction of ampicillin, a worsening after switching to clindamycin and azithromycin is most likely caused by a DHR to ampicillin alone and not because of additional DHRs to the other drugs.

Another important step in risk assessment is the evaluation of the role of the drugs given using lists of the typical elicitors of specific allergic reactions. For example, if a benign drug exanthema has developed and amoxicillin, thyroxine and propanolol have been newly introduced about 1 week before the beginning of the exanthem, amoxicillin is by far the most likely elicitor and should be stopped, whereas the minimal risk for the other drugs to have caused the reaction can be balanced against the need to continue this medication. For benign exanthemas, penicillins, especially aminopenicillins (ampicillin, amoxicillin), are the most common elicitors, followed by cephalosporins, sulfonamide antibiotics, macrolides, allopurinol, and antiepileptics, whereas for urticaria and anaphylaxis, NSAIDs, penicillins, cephalosporins, NMBA, ICM and proton pump blockers are the most common culprits.

If the intake of a suspected drug is stopped but there is continued need for drug therapy, alternative medications with minimal or no increased risk of reaction should be given. In order to do so, structural similarities between the culprit and the newly given drug should be avoided.

Cross-reactivity is based on the similarity of drug structure between drug groups and is particularly important for BLs, NSAIDs, ICM and NMBA [9, 52–54]. There is a debate as to whether antibacterial sulfonamides cross-react with other sulfonamides. However, with the exception of DRESS, where immune deviation may lead to a broadening of sensitivity to other less similar drugs, switching to a totally different drug class does not carry increased risk for a reaction. For example, in patients with BL allergy, non-BL antibiotics may be given.

**Fig. 3:**
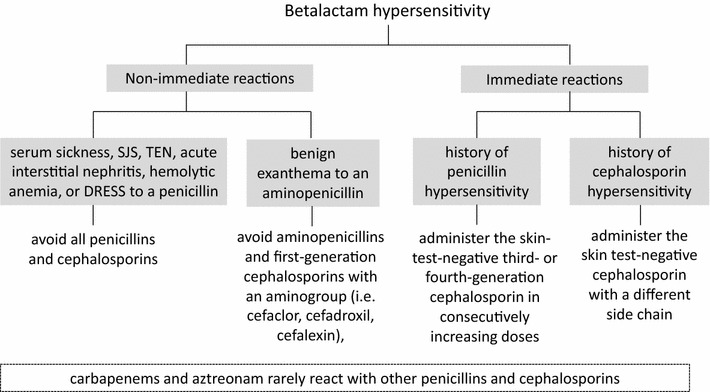
Management and alternatives for BL hypersensitive patients

Even in patients with BL allergy, specific IgE is mostly directed against a specific side chain of the drug and not the central betalactam ring [52]. Patient with a history of serum sickness, SJS, TEN, acute interstitial nephritis, hemolytic anemia, or DRESS after intake of BLs should generally avoid all penicillins and cephalosporins because of the severity of the reported reaction [48]. However, patients with common benign exanthema to an aminopenicillin (ampicillin or amoxicillin) do generally tolerate all other non-aminopenicillins and cephalosporins, other than those first-generation preparations with an amino group (e.g. cefaclor, cefadroxil, cefalexin), and giving these other BLs can be considered if urgent, when a proper drug allergy diagnostic procedure is not possible [55]. The same holds true for carbapenems and aztreonam, which rarely react with other penicillins and cephalosporins. It should be noted that the increased risk concerns not an immediate reaction, such as anaphylaxis, but that of a renewed exanthema (Fig. 3).

For immediate reactions, recommendations are more difficult, because severe and sometimes fatal reactions may arise if a drug cross-reacts. However, cross-reactivity between penicillins and third- or fourth-generation cephalosporins or carbapenems are very rare [56]. In the case of an urgent need, without the possibility of complete allergological testing in a patient with a history of DHR to penicillin, it would be best to perform a skin test including intradermal test with the test concentrations recommended and to administer the alternative skin-test-negative third- or fourth-generation cephalosporin or carbapenems or aztreonam in consecutive increasing doses (e.g. 1/10 followed by 1/2 and 1/1 of a typical simple dose in 2-h intervals) [46]. Even without skin testing the risk would be low in this situation, but a risk–benefit analysis would have to be made and emergency preparedness is necessary. A similar approach can be taken for a patient with a history of DHRs to cephalosporin, where a skin test-negative cephalosporin with a different side chain can be selected for therapy with only minimal risk of a severe reaction [57].

Concerning NSAIDs, patients with immunologically mediated hypersensitivity reactions should avoid the culprit drug and chemically related drugs, whilst being able to take other NSAIDs that are not chemically related. However, non–immunologically mediated hypersensitivity reactions to NSAIDs are more frequent and cross-reactivity exists between cyclooxygenase (COX)-1 enzyme inhibitory drugs and between pyrazolones (metamizole, propyphenazone) [9]. Thus, if a patient who has reacted with urticaria to acetylsalicylic acid needs another NSAID, he is very likely to also react to e.g. ibuprofen. Skin tests are of value for pyrazolones only, and do not help in the field of COX-1 inhibitor cross-reactivity. If a patient with a previous reaction to a COX-1 NSAID is in urgent need of pain medication and allergy testing cannot be arranged, he will most likely tolerate opioids because of their very different structure. In addition, selective COX-2 inhibitors are tolerated by the vast majority of these patients and can also be considered to be given in emergencies. It should be stated again that allergological testing in the symptom-free interval is preferable to giving these drugs in urgent need where a risk–benefit analysis is necessary.

Proper DHR documentation given to the patient is crucial to prevent accidental future exposure to culprit drugs [58]. It is important to provide the patient with written information in the form of a letter or certificate and it is also recommended that they wear a bracelet or similar, indicating the drugs they are allergic to. All healthcare professionals issuing or handing out prescriptions or drugs should be informed which drugs or drug classes should be avoided, which reactions occurred in the history of the patient, which test procedures have been performed, and what the results of these test procedures were. The patient and their family members or caregivers should be aware of the drugs or drug classes that they need to avoid, moreover they should present the written information to any prescribing doctor or pharmacist before obtaining a drug. Thus, patients should carry this information at all times and should share it with all healthcare professionals. Lack of proper drug hypersensitivity documentation is a common reason for prescription errors. The referral to an allergist for the assessment of DHRs is recommended during a symptom-free interval.

### Unmet needs and research

The diagnosis of DHRs is a complex issue. The first step is an accurate identification of whether the patient has a suspected DHR. Any symptom that appears in the context of drug-intake is usually described by patients as an allergy to that drug. Many patients have never heard of DHRs or ADRs, therefore they would seldom use this terminology. The identification of whether the patient has a suspected DHR should be performed by primary care physicians, who are generally the first practitioners the patient consults with, although this can also apply to other non-allergist physicians. Therefore, these physicians play an important role in deciding whether a patient needs to be sent to the allergist. They are also responsible for prescribing a safe alternative. However, there is currently a critical lack of skills and knowledge in the field of DHRs.

Pre- and post-graduate allergy educational programs and training should be implemented. Primary care physicians should recieve training in specific approaches for the diagnosis and management of ADRs, enabling them to recognize the reaction, to identify severe reactions that require urgent management, to stop the medication suspected to have caused the reaction, and to give a non-cross-reactive alternative drug. However, currently there is no minimum data set of requirements or standardized format which can be used to record an ADR: this is an important unmet area of research that needs to be developed. Moreover, in most European countries, primary care guidelines and pathways about drug hypersensitivity testing are not adequately addressed, and need further investigation. Primary care physicians should also be trained in criteria for referring patients experiencing DHRs to specialists. There should also be increased awareness for the recognition and management of DHRs. It is important that primary care physicians provide the patient with written information, however there is no standardized format for this either, and further research is required to inform this process. Moreover, a close relationship is necessary between primary care physicians and specialists involved in the diagnosis of patients with drug allergy in order to give the best care to patients. Therefore, the implementation of better and standardized care pathways, facilitating closer communication between primary care physicians and specialists is essential. Moreover, referral times should be rapid, in order to avoid problems related to patient recall and test negativization rates, as described above. This depends on convinving commissioners and others responsible for the service, who need to understand that this is a cost effective way of providing high quality care. Crucially, when a patient thought to suffer from DHRs is proven not to react to a given drug, their records should be updated appropriately, to reduce the problem of overdiagnosis. The implementation of these measures is needed to strengthen the crucial role that primary care physicians have in diagnosing and managing DHRs.

## Conclusions

The primary care physician plays a key role in identifying which patients may have suffered a DHR as they are generally the first practitioners the patient consults with. They are responsible for referring the patient to the specialist for further allergological testing and for prescribing a safe alternative until the diagnosis is finally confirmed. However, there is currently a critical lack of knowledge in the field of DHRs that has led to recognised deficits in managing DHRs. There is a clear need for educational programmes for training primary care physician in specific approaches for the diagnosis and management of ADRs.

## Abbreviations

AGEP: acute generalized exanthematous pustulosis
ADRs: adverse drug reactions,
BATs: basophils activation tests
BL: betalactam
CSU: chronic spontaneous urticaria
COX: cyclooxygenase
DHRs: drug hypersensitivity reactions
DHIS: drug-induced hypersensitivity syndrome
DPTs: drug provocation tests
DRESS: drug reaction with eosinophilia and systemic symptoms
IDTs: intradermal tests
ICM: iodinated contrast media
NMBA: neuromuscular blocking agents
NSAIDs: non-steroidal anti-inflammatory drugs
SJS: Stevens–Johnson syndrome
SDRIFE: symmetric drug-related intertriginous and flexural exanthemas
TEN: toxic epidermal necrolysis
